# Supplementary material for: A Likelihood Approach for Real-Time Calibration of Stochastic Compartmental Epidemic Models
Source: PLoS Comput Biol. 2017 Jan 17;13(1):e1005257. doi: 10.1371/journal.pcbi.1005257 (PMC5240920; doi:10.1371/journal.pcbi.1005257)
Supplement: S1 File — (TAR.GZ) [file pcbi.1005257.s014.tar.gz › HSPH_Online-SI-Revision/IPoi09/n100-severe/n100-severe_BENCH2_table8.pdf]

1	newly 3 weeks spec	405.	1454.25	{748., 2262.}	259.074	0.476178	newly 3 weeks cumul	1885.	5708.82	{2998., 8781.}	202.855	0.41169	newly 5 weeks spec	212.	856.983	{438., 1242.}	304.237	0.521636	newly 5 weeks cumul	2436.	7692.7	{4003., 11 655.}	215.792	0.426327	newly 42 weeks cumul	2940.	10 443.9	{5268., 15 591.}	255.233	0.472456
2	newly 3 weeks spec	32.	136.984	{46., 209.}	328.075	0.510079	newly 3 weeks cumul	144.	756.548	{292., 1130.}	425.381	0.606783	newly 5 weeks spec	7.	47.3871	{17., 64.}	576.959	0.711628	newly 5 weeks cumul	163.	886.065	{336., 1282.}	443.598	0.621533	newly 49 weeks cumul	163.	954.839	{362., 1383.}	485.791	0.653931
3	newly 3 weeks spec	20.	114.852	{56., 232.}	474.262	0.683785	newly 3 weeks cumul	87.	633.918	{321., 1274.}	628.641	0.790138	newly 5 weeks spec	9.	41.5246	{19., 84.}	361.384	0.588767	newly 5 weeks cumul	101.	744.541	{376., 1478.}	637.169	0.796048	newly 50 weeks cumul	112.	806.82	{406., 1602.}	620.375	0.786676
4	newly 3 weeks spec	173.	317.79	{162., 643.}	87.4417	0.21309	newly 3 weeks cumul	880.	1476.06	{791., 2864.}	73.9003	0.18824	newly 5 weeks spec	105.	138.903	{70., 270.}	55.5453	0.171516	newly 5 weeks cumul	1112.	1826.97	{962., 3545.}	71.7162	0.184488	newly 48 weeks cumul	1341.	2111.23	{1125., 4034.}	67.066	0.176911
5	newly 3 weeks spec	780.	1182.23	{539., 1929.}	66.9777	0.18289	newly 3 weeks cumul	2856.	4695.72	{2209., 7548.}	74.624	0.195437	newly 5 weeks spec	402.	697.639	{325., 1112.}	84.1367	0.21014	newly 5 weeks cumul	3744.	6302.57	{2978., 10 043.}	77.871	0.199831	newly 42 weeks cumul	4829.	8483.77	{3970., 13 494.}	83.9949	0.209406
6	newly 3 weeks spec	252.	334.967	{154., 519.}	58.1772	0.1787	newly 3 weeks cumul	1205.	1569.85	{679., 2431.}	57.5009	0.181092	newly 5 weeks spec	97.	144.049	{63., 225.}	66.385	0.184709	newly 5 weeks cumul	1441.	1932.57	{836., 2989.}	59.2098	0.181099	newly 48 weeks cumul	1575.	2216.3	{965., 3422.}	62.421	0.181874
7	newly 3 weeks spec	63.	324.1	{166., 487.}	414.444	0.64376	newly 3 weeks cumul	435.	1620.73	{853., 2365.}	272.582	0.50586	newly 5 weeks spec	20.	133.2	{68., 184.}	566.	0.757905	newly 5 weeks cumul	497.	1957.82	{1017., 2828.}	293.927	0.530224	newly 49 weeks cumul	509.	2183.8	{1127., 3166.}	329.037	0.5664
8	newly 3 weeks spec	292.	1000.62	{440., 1814.}	242.679	0.458368	newly 3 weeks cumul	1253.	4211.36	{1943., 7728.}	236.102	0.452979	newly 5 weeks spec	140.	526.197	{246., 917.}	275.855	0.495788	newly 5 weeks cumul	1606.	5465.8	{2544., 9902.}	240.336	0.45737	newly 45 weeks cumul	1911.	6842.23	{3115., 12 417.}	258.044	0.477965
9	newly 3 weeks spec	0.1	168.017	{58., 323.}	167 917.	3.10326	newly 3 weeks cumul	4.	949.733	{308., 1828.}	23 643.3	2.25535	newly 5 weeks spec	0.1	57.1	{19., 87.}	57 000.	2.6314	newly 5 weeks cumul	4.	1103.2	{371., 2105.}	27 480.	2.32051	newly 50 weeks cumul	4.	1181.4	{393., 2204.}	29 435.	2.35152
10	newly 3 weeks spec	115.	1228.71	{552., 2142.}	968.443	0.943576	newly 3 weeks cumul	507.	4985.71	{2239., 8742.}	883.375	0.914135	newly 5 weeks spec	26.	683.742	{302., 1248.}	2529.78	1.32743	newly 5 weeks cumul	597.	6586.73	{2959., 11 609.}	1003.3	0.961505	newly 43 weeks cumul	631.	8560.76	{3763., 15 065.}	1256.7	1.04826
11	newly 3 weeks spec	53.	286.258	{144., 472.}	440.11	0.666145	newly 3 weeks cumul	267.	1426.31	{724., 2259.}	434.197	0.663632	newly 5 weeks spec	7.	119.597	{59., 182.}	1608.53	1.16462	newly 5 weeks cumul	288.	1727.47	{889., 2730.}	499.815	0.713648	newly 49 weeks cumul	300.	1936.55	{996., 3083.}	545.516	0.745574
12	newly 3 weeks spec	467.	1421.83	{703., 2262.}	204.46	0.402012	newly 3 weeks cumul	2017.	5584.29	{2717., 8781.}	176.861	0.370717	newly 5 weeks spec	256.	837.339	{373., 1242.}	227.086	0.42651	newly 5 weeks cumul	2654.	7524.71	{3599., 11 655.}	183.523	0.3773	newly 42 weeks cumul	3292.	10 211.8	{4805., 15 591.}	210.199	0.411128
13	newly 3 weeks spec	476.	1182.23	{539., 1929.}	148.719	0.319377	newly 3 weeks cumul	2172.	4695.72	{2209., 7548.}	118.154	0.272072	newly 5 weeks spec	224.	697.639	{325., 1112.}	211.446	0.4107	newly 5 weeks cumul	2758.	6302.57	{2978., 10 043.}	129.699	0.290259	newly 42 weeks cumul	3330.	8483.77	{3970., 13 494.}	154.93	0.330413
14	newly 3 weeks spec	744.	1324.83	{589., 1878.}	86.8638	0.188492	newly 3 weeks cumul	3264.	5070.97	{2364., 7671.}	69.421	0.17412	newly 5 weeks spec	407.	825.083	{336., 1113.}	108.784	0.210605	newly 5 weeks cumul	4260.	6954.33	{3137., 10 358.}	75.6768	0.178687	newly 42 weeks cumul	5829.	9683.3	{4197., 14 192.}	79.8776	0.180638
15	newly 3 weeks spec	170.	742.113	{290., 1179.}	336.537	0.540685	newly 3 weeks cumul	891.	3177.16	{1299., 5083.}	256.584	0.460092	newly 5 weeks spec	60.	386.242	{160., 600.}	543.737	0.706608	newly 5 weeks cumul	1068.	4095.74	{1643., 6545.}	283.496	0.490117	newly 44 weeks cumul	1164.	5048.89	{2065., 8105.}	333.753	0.541947
16	newly 3 weeks spec	648.	1436.44	{619., 2172.}	124.559	0.279287	newly 3 weeks cumul	2744.	5656.76	{2604., 8799.}	109.392	0.258897	newly 5 weeks spec	394.	857.175	{373., 1346.}	121.578	0.270215	newly 5 weeks cumul	3600.	7634.6	{3425., 11 829.}	115.104	0.266306	newly 43 weeks cumul	4481.	10 354.5	{4434., 15 961.}	132.869	0.292659
17	newly 3 weeks spec	33.	278.629	{106., 429.}	744.33	0.801191	newly 3 weeks cumul	309.	1473.85	{541., 2185.}	376.976	0.553267	newly 5 weeks spec	37.	106.371	{39., 169.}	190.715	0.349762	newly 5 weeks cumul	396.	1754.06	{649., 2627.}	342.946	0.521252	newly 49 weeks cumul	405.	1917.4	{712., 2842.}	373.433	0.550315
18	newly 3 weeks spec	122.	460.557	{166., 976.}	278.178	0.43101	newly 3 weeks cumul	687.	2198.13	{778., 4770.}	221.416	0.367522	newly 5 weeks spec	37.	194.049	{64., 413.}	424.457	0.559146	newly 5 weeks cumul	830.	2687.92	{918., 5881.}	225.129	0.369918	newly 47 weeks cumul	928.	3050.51	{1058., 6663.}	229.757	0.373868
19	newly 3 weeks spec	77.	334.967	{154., 519.}	335.022	0.558248	newly 3 weeks cumul	381.	1569.85	{679., 2431.}	312.035	0.533694	newly 5 weeks spec	32.	144.049	{63., 225.}	350.154	0.570816	newly 5 weeks cumul	445.	1932.57	{836., 2989.}	334.286	0.556219	newly 47 weeks cumul	467.	2216.3	{965., 3422.}	374.581	0.59427
20	newly 3 weeks spec	99.	883.082	{437., 1373.}	792.002	0.880302	newly 3 weeks cumul	531.	3741.51	{1849., 5770.}	604.615	0.780476	newly 5 weeks spec	19.	464.672	{221., 699.}	2345.64	1.31491	newly 5 weeks cumul	601.	4843.67	{2391., 7417.}	705.935	0.838006	newly 45 weeks cumul	622.	6030.61	{2946., 9132.}	869.551	0.917131
21	newly 3 weeks spec	24.	203.883	{99., 283.}	749.514	0.862681	newly 3 weeks cumul	229.	1032.03	{533., 1479.}	350.67	0.587441	newly 5 weeks spec	14.	82.3333	{36., 121.}	488.095	0.688475	newly 5 weeks cumul	254.	1247.57	{634., 1786.}	391.168	0.624552	newly 49 weeks cumul	256.	1391.22	{711., 2005.}	443.444	0.667768
22	newly 3 weeks spec	57.	497.803	{208., 663.}	773.339	0.809577	newly 3 weeks cumul	329.	2319.26	{927., 3153.}	604.943	0.722573	newly 5 weeks spec	27.	223.475	{92., 319.}	727.687	0.774473	newly 5 weeks cumul	392.	2880.1	{1150., 3957.}	634.719	0.737426	newly 47 weeks cumul	426.	3331.8	{1322., 4484.}	682.113	0.762814
23	newly 3 weeks spec	0.1	56.1	{23., 72.}	56 000.	2.66067	newly 3 weeks cumul	12.	345.167	{160., 494.}	2776.39	1.37614	newly 5 weeks spec	0.1	17.	{7., 26.}	16 900.	2.12282	newly 5 weeks cumul	12.	391.167	{181., 571.}	3159.72	1.43075	newly 51 weeks cumul	12.	410.517	{195., 596.}	3320.97	1.453
24	newly 3 weeks spec	10.	80.3387	{41., 113.}	703.387	0.836989	newly 3 weeks cumul	103.	487.387	{256., 701.}	373.191	0.611151	newly 5 weeks spec	0.1	24.2097	{11., 38.}	24 109.7	2.30808	newly 5 weeks cumul	103.	556.532	{295., 797.}	440.323	0.669016	newly 52 weeks cumul	103.	587.226	{310., 842.}	470.122	0.692205
25	newly 3 weeks spec	0.1	46.3651	{16., 75.}	46 265.1	2.5499	newly 3 weeks cumul	33.	320.81	{130., 512.}	872.15	0.881628	newly 5 weeks spec	0.1	12.2857	{3., 22.}	12 185.7	1.9363	newly 5 weeks cumul	33.	357.571	{146., 597.}	983.55	0.927332	newly 52 weeks cumul	33.	371.111	{152., 620.}	1024.58	0.943029
26	newly 3 weeks spec	105.	743.783	{308., 1389.}	608.365	0.766445	newly 3 weeks cumul	559.	3310.35	{1382., 6108.}	492.191	0.691115	newly 5 weeks spec	34.	364.7	{152., 682.}	972.647	0.945598	newly 5 weeks cumul	657.	4202.75	{1811., 7746.}	539.688	0.723856	newly 46 weeks cumul	704.	5046.98	{2165., 9310.}	616.901	0.772446
27	newly 3 weeks spec	67.	457.656	{210., 687.}	583.068	0.757906	newly 3 weeks cumul	373.	2137.05	{1068., 3190.}	472.935	0.685553	newly 5 weeks spec	19.	208.131	{93., 320.}	995.427	0.964436	newly 5 weeks cumul	421.	2651.69	{1315., 3923.}	529.855	0.726467	newly 47 weeks cumul	444.	3080.38	{1510., 4564.}	593.779	0.768356
28	newly 3 weeks spec	219.	571.683	{237., 1287.}	161.811	0.314528	newly 3 weeks cumul	956.	2599.24	{1076., 5817.}	172.146	0.333357	newly 5 weeks spec	119.	263.095	{107., 613.}	125.997	0.258128	newly 5 weeks cumul	1227.	3248.94	{1346., 7317.}	165.18	0.32146	newly 46 weeks cumul	1619.	3805.06	{1532., 8481.}	137.547	0.279398
29	newly 3 weeks spec	122.	457.656	{210., 687.}	275.128	0.497621	newly 3 weeks cumul	698.	2137.05	{1068., 3190.}	206.168	0.413407	newly 5 weeks spec	37.	208.131	{93., 320.}	462.517	0.674988	newly 5 weeks cumul	790.	2651.69	{1315., 3923.}	235.657	0.453122	newly 47 weeks cumul	845.	3080.38	{1510., 4564.}	264.542	0.488883
30	newly 3 weeks spec	189.	1083.15	{521., 1712.}	473.094	0.67627	newly 3 weeks cumul	1013.	4455.92	{2230., 7115.}	339.873	0.567642	newly 5 weeks spec	86.	587.459	{288., 904.}	583.092	0.74477	newly 5 weeks cumul	1247.	5844.57	{2872., 9262.}	368.691	0.592544	newly 43 weeks cumul	1455.	7429.25	{3553., 11 648.}	410.601	0.626529
31	newly 3 weeks spec	1.	12.8475	{5., 19.}	1184.75	1.00222	newly 3 weeks cumul	19.	93.6441	{41., 156.}	392.864	0.612222	newly 5 weeks spec	0.1	3.27119	{0., 6.}	3198.31	1.28123	newly 5 weeks cumul	19.	103.339	{46., 178.}	443.889	0.656704	newly 52 weeks cumul	19.	106.373	{49., 183.}	459.857	0.670888
32	newly 3 weeks spec	28.	148.258	{75., 210.}	429.493	0.660333	newly 3 weeks cumul	154.	826.661	{398., 1204.}	436.793	0.667331	newly 5 weeks spec	6.	50.9032	{25., 76.}	748.387	0.865144	newly 5 weeks cumul	176.	968.081	{472., 1388.}	450.046	0.678179	newly 50 weeks cumul	190.	1045.53	{506., 1494.}	450.28	0.678447
33	newly 3 weeks spec	32.	114.852	{56., 232.}	258.914	0.479665	newly 3 weeks cumul	204.	633.918	{321., 1274.}	210.744	0.420027	newly 5 weeks spec	18.	41.5246	{19., 84.}	134.335	0.307548	newly 5 weeks cumul	235.	744.541	{376., 1478.}	216.826	0.429302	newly 50 weeks cumul	268.	806.82	{406., 1602.}	201.052	0.407759
34	newly 3 weeks spec	218.	1454.25	{748., 2262.}	567.087	0.745177	newly 3 weeks cumul	1079.	5708.82	{2998., 8781.}	429.084	0.65398	newly 5 weeks spec	118.	856.983	{438., 1242.}	626.257	0.776089	newly 5 weeks cumul	1346.	7692.7	{4003., 11 655.}	471.523	0.683959	newly 42 weeks cumul	1517.	10 443.9	{5268., 15 591.}	588.454	0.759818
35	newly 3 weeks spec	0.1	63.8387	{30., 107.}	63 738.7	2.73955	newly 3 weeks cumul	24.	401.726	{186., 625.}	1573.86	1.15621	newly 5 weeks spec	0.1	19.3387	{8., 36.}	19 238.7	2.20827	newly 5 weeks cumul	24.	456.048	{218., 733.}	1800.2	1.21188	newly 51 weeks cumul	24.	482.065	{235., 781.}	1908.6	1.23658
36	newly 3 weeks spec	53.	523.262	{217., 864.}	887.287	0.898732	newly 3 weeks cumul	336.	2544.11	{1090., 4215.}	657.177	0.788645	newly 5 weeks spec	17.	222.393	{89., 362.}	1208.2	1.01651	newly 5 weeks cumul	377.	3108.34	{1333., 5145.}	724.494	0.824549	newly 49 weeks cumul	391.	3524.95	{1518., 5815.}	801.522	0.863048
37	newly 3 weeks spec	3.	50.0159	{21., 96.}	1567.2	1.12833	newly 3 weeks cumul	35.	320.984	{143., 574.}	817.098	0.868645	newly 5 weeks spec	0.1	15.2381	{4., 27.}	15 141.3	2.02531	newly 5 weeks cumul	35.	363.333	{156., 683.}	938.095	0.922976	newly 51 weeks cumul	35.	379.54	{161., 723.}	984.399	0.942186
38	newly 3 weeks spec	40.	286.258	{144., 472.}	615.645	0.78836	newly 3 weeks cumul	256.	1426.31	{724., 2259.}	457.151	0.681903	newly 5 weeks spec	12.	119.597	{59., 182.}	896.64	0.930534	newly 5 weeks cumul	282.	1727.47	{889., 2730.}	512.577	0.722792	newly 49 weeks cumul	298.	1936.55	{996., 3083.}	549.848	0.748479
39	newly 3 weeks spec	150.	270.552	{99., 519.}	97.4627	0.240259	newly 3 weeks cumul	734.	1306.18	{452., 2461.}	96.287	0.241812	newly 5 weeks spec	40.	121.328	{45., 226.}	205.634	0.385747	newly 5 weeks cumul	880.	1608.97	{582., 3060.}	100.053	0.24664	newly 46 weeks cumul	975.	1842.93	{652., 3500.}	104.96	0.252832
40	newly 3 weeks spec	92.	256.127	{64., 510.}	190.494	0.319882	newly 3 weeks cumul	452.	1395.07	{355., 2618.}	216.199	0.346291	newly 5 weeks spec	39.	89.7636	{22., 161.}	153.66	0.290132	newly 5 weeks cumul	559.	1639.78	{443., 3068.}	202.072	0.329042	newly 49 weeks cumul	609.	1770.69	{480., 3284.}	199.442	0.325069
41	newly 3 weeks spec	14.	56.1	{23., 72.}	300.714	0.514538	newly 3 weeks cumul	127.	345.167	{160., 494.}	172.546	0.35503	newly 5 weeks spec	4.	17.	{7., 26.}	327.5	0.534963	newly 5 weeks cumul	135.	391.167	{181., 571.}	190.025	0.380827	newly 51 weeks cumul	136.	410.517	{195., 596.}	201.9	0.398855
42	newly 3 weeks spec	202.	1254.17	{531., 2888.}	520.876	0.703912	newly 3 weeks cumul	987.	5082.63	{2231., 11 663.}	414.957	0.628885	newly 5 weeks spec	95.	702.576	{300., 1645.}	639.554	0.773519	newly 5 weeks cumul	1233.	6727.02	{2918., 15 499.}	445.581	0.651678	newly 44 weeks cumul	1344.	8739.68	{3802., 20 181.}	550.274	0.725086
43	newly 3 weeks spec	252.	908.582	{316., 1645.}	260.952	0.43645	newly 3 weeks cumul	1153.	3791.56	{1338., 6988.}	229.49	0.410565	newly 5 weeks spec	99.	474.018	{159., 867.}	378.806	0.540407	newly 5 weeks cumul	1387.	4911.67	{1718., 9089.}	254.35	0.435808	newly 43 weeks cumul	1536.	6062.55	{2089., 11 057.}	294.697	0.476639
44	newly 3 weeks spec	39.	225.754	{115., 431.}	478.857	0.703028	newly 3 weeks cumul	267.	1171.64	{602., 2288.}	338.816	0.584876	newly 5 weeks spec	9.	83.9016	{42., 161.}	832.24	0.902937	newly 5 weeks cumul	301.	1392.67	{720., 2726.}	362.682	0.607813	newly 50 weeks cumul	304.	1524.85	{793., 2990.}	401.596	0.642973
45	newly 3 weeks spec	130.	495.607	{213., 773.}	281.236	0.498138	newly 3 weeks cumul	816.	2352.34	{1041., 3588.}	188.277	0.379333	newly 5 weeks spec	64.	218.672	{94., 360.}	241.675	0.447164	newly 5 weeks cumul	987.	2905.98	{1291., 4440.}	194.426	0.38833	newly 48 weeks cumul	1048.	3333.75	{1486., 5098.}	218.106	0.421573
46	newly 3 weeks spec	72.	876.323	{413., 1573.}	1117.11	1.0025	newly 3 weeks cumul	478.	3827.18	{1745., 6971.}	700.665	0.825387	newly 5 weeks spec	30.	430.5	{202., 800.}	1335.	1.06727	newly 5 weeks cumul	550.	4880.9	{2249., 8837.}	787.437	0.868371	newly 47 weeks cumul	566.	5907.39	{2699., 10 744.}	943.708	0.937167
47	newly 3 weeks spec	463.	1078.4	{472., 1788.}	134.345	0.290748	newly 3 weeks cumul	1902.	4354.6	{1952., 7395.}	130.319	0.287438	newly 5 weeks spec	209.	605.839	{259., 1059.}	189.875	0.375464	newly 5 weeks cumul	2424.	5771.74	{2552., 9827.}	139.074	0.300756	newly 43 weeks cumul	2941.	7584.81	{3300., 12 911.}	158.312	0.330153
48	newly 3 weeks spec	166.	495.607	{213., 773.}	198.558	0.391974	newly 3 weeks cumul	968.	2352.34	{1041., 3588.}	144.054	0.309927	newly 5 weeks spec	51.	218.672	{94., 360.}	328.769	0.545774	newly 5 weeks cumul	1120.	2905.98	{1291., 4440.}	159.802	0.334969	newly 48 weeks cumul	1215.	3333.75	{1486., 5098.}	174.515	0.357944
49	newly 3 weeks spec	3.	40.	{20., 64.}	1233.33	1.05173	newly 3 weeks cumul	31.	251.597	{123., 349.}	711.602	0.845549	newly 5 weeks spec	0.1	11.2097	{4., 20.}	11 109.7	1.9577	newly 5 weeks cumul	32.	283.919	{143., 395.}	787.248	0.884622	newly 52 weeks cumul	32.	296.113	{152., 418.}	825.353	0.90351
50	newly 3 weeks spec	18.	56.1	{23., 72.}	211.852	0.406221	newly 3 weeks cumul	46.	345.167	{160., 494.}	650.362	0.792565	newly 5 weeks spec	1.	17.	{7., 26.}	1600.	1.12282	newly 5 weeks cumul	52.	391.167	{181., 571.}	652.244	0.793927	newly 51 weeks cumul	52.	410.517	{195., 596.}	689.455	0.816176
